# Integration of time-resolved transcriptomics data with flux-based methods reveals stress-induced metabolic adaptation in *Escherichia coli*

**DOI:** 10.1186/1752-0509-6-148

**Published:** 2012-11-30

**Authors:** Nadine Töpfer, Szymon Jozefczuk, Zoran Nikoloski

**Affiliations:** 1 Systems Biology and Mathematical Modeling Group, Max-Planck-Institute of Molecular Plant Physiology, 14476 Potsdam, Germany; 2 ETH Zurich, Institute of Molecular Systems Biology, 8093 Zurich, Switzerland

**Keywords:** Flux-based methods, Genome-scale metabolic network, Network optimization, Adaptation

## Abstract

**Background:**

Changes in environmental conditions require temporal effectuation of different metabolic pathways in order to maintain the organisms’ viability but also to enable the settling into newly arising conditions. While analyses of robustness in biological systems have resulted in the characterization of reactions that facilitate homeostasis, temporal adaptation-related processes and the role of cellular pathways in the metabolic response to changing conditions remain elusive.

**Results:**

Here we develop a flux-based approach that allows the integration of time-resolved transcriptomics data with genome-scale metabolic networks. Our framework uses bilevel optimization to extract temporal minimal operating networks from a given large-scale metabolic model. The minimality of the extracted networks enables the computation of elementary flux modes for each time point, which are in turn used to characterize the transitional behavior of the network as well as of individual reactions. Application of the approach to the metabolic network of *Escherichia coli* in conjunction with time-series gene expression data from cold and heat stress results in two distinct time-resolved modes for reaction utilization—constantly active and temporally (de)activated reactions. These patterns contrast the processes for the maintenance of basic cellular functioning and those required for adaptation. They also allow the prediction of reactions involved in time- and stress-specific metabolic response and are verified with respect to existing experimental studies.

**Conclusions:**

Altogether, our findings pinpoint the inherent relation between the systemic properties of robustness and adaptability arising from the interplay of metabolic network structure and changing environment.

## Background

The steady-state metabolism of microorganisms has evolved to optimize growth under ambient conditions [1]. However, under suboptimal conditions or upon perturbation, organisms must maintain homeostasis and adapt their modes of operation to ensure viability [2]. Maintenance of homeostasis has already been addressed in the context of studying system’s robustness [3,4]. The underlying mechanisms stabilize a cellular function under changing conditions and often involve feedback control [5,6]. In turn, adaptability refers to adjustment in systemic properties (*e.g.*, utilization of available nutrients) in order to facilitate the transition between conditions. The two properties—robustness and adaptability—do not exclude each other since both arise from the necessity of an organism to cope with its environment.

While robustness has been widely studied [4,7], (metabolic) adaptability has not been systematically investigated, largely due to the lack of a precise formulation and its global effects on the organism. Therefore, any approach to capture and analyze adaptation-related processes requires the consideration of a comprehensive network of metabolic pathways in order to capture the complex interplay of network constituents.

Several approaches that integrate data with graph-theoretic methods have been applied to obtain subnetworks engaged under different conditions. For instance, [8] uses transcriptomics data in combination with protein-protein interaction networks to identify active subnetworks that show levels in differential expression for particular subsets of conditions. However, graph-theoretic approaches neglect the stoichiometry of the considered biochemical reactions. Thus, it is difficult to relate the findings from these approaches to network functionality and growth.

With the increasing availability and quality of genome-scale metabolic models and high-throughput data, constraint-based methods that integrate these data have found broad applications. For instance, a genome-scale metabolic model has been coupled with transcriptomics data, based on Boolean logic, to improve flux predictions [9]. Thereby, a flux is constrained to zero, if the respective transcript has not been observed. Another attempt employs transcriptomics and proteomics data to derive tissue-specific metabolic activity [10] and is based on a trivalued logic to maximize the number of reactions in the network that are consistent with the expression data. To overcome the issue of selecting an arbitrary threshold value in considering a gene “on” or “off”, a method, referred to as MADE, was proposed. It employs the statistical significance of changes in gene or protein expression data between two cellular states to extract metabolic models (subnetworks) that reflect the expression dynamics [11].

While constraint-based methods usually provide solutions that optimize a certain objective, elementary flux modes (EFMs) capture the whole spectrum of metabolic steady states of a given network. An EFM is defined as a minimal set of reactions that can operate at steady state [12]. EFM-based analysis have been applied to study robustness [13] and explore structural properties of new pathways [14]. Although promising attempts for enumerating subsets of EFMs, identifying pathways in genome-scale metabolic networks [15,16], as well as for sampling a given number of EFMs [17] have been proposed, the problem of combinatorial explosion restrains the computation of EFMs to networks of moderate size [18].

Flux-based, *i.e.*, constraint- and EFM-based, approaches have proven useful in characterizing stationary metabolic states of an organism. However, the adaptation of metabolism to changing conditions is a temporal process, and the state of the organism strongly depends on the time scale after the perturbation. Therefore, in order to capture adaptation-related processes, it is necessary to develop and apply a computational method which allows the integration of time-series data, uses the advantages of flux-based methods, and overcomes some of the shortcomings of the briefly reviewed approaches.

**Figure 1:**
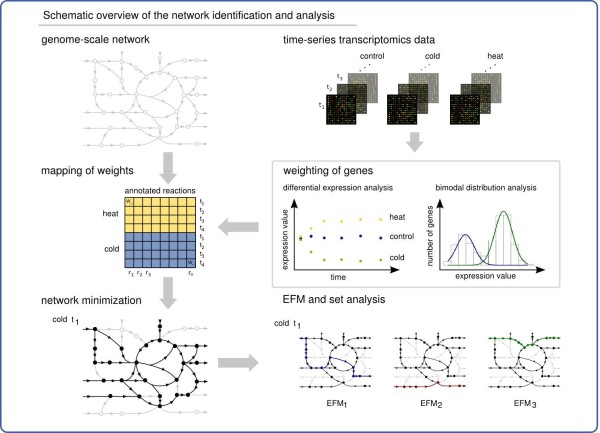
**Schematic depiction of the computational approach.** A genome-scale network and time-series transcriptomics data are used to extract time- and condition-specific minimal networks. Data for different environmental conditions are analyzed to weight genes based on differential expression and bimodal distribution analyses. The gene-reaction annotation of the network reconstruction is used to map the weights onto the metabolic model. A minimization approach is applied to extract minimal networks. EFM analysis is conducted on the minimal networks, and the resulting sets of EFMs and the derived fractional appearance profiles are employed to characterize the transitional behavior of the network and of individual reactions, respectively.

Here we present a novel method, which we term Adaptation of Metabolism (AdaM), to identify reactions and pathways that enable system adaptation upon external perturbation. AdaM integrates time-series transcriptomics data with flux-based bilevel optimization to extract minimal operating networks from a given large-scale metabolic model. The minimality of the extracted networks enables the computation of EFMs for each time point. These sets of EFMs are in turn used to characterize the transitional behavior of the network as well as of individual reactions (see Figure 1). The theoretical framework is applied to recently obtained transcriptomics data for cold and heat stress from *E. coli*[19] and is compared to MADE. Our findings reveal differences in response patterns for the two investigated conditions and characterize (de)activation patterns associated to temperature stress. The model-based and data-driven predictions are verified with respect to results from the existing experimental studies. Finally, our results are used to posit novel hypotheses related to temperature-associated metabolic adaptation processes.

## Methods

### Weighting of reactions

Transcriptomics data are used to weight reactions that are catalyzed by the enzymes encoded in the respective genes. To determine pathways that contribute to the metabolic state of the organism, we consider reactions, that are not only temporally activated upon the changed environmental condition, but also reactions that are constantly active. Therefore, we combine information obtained from the analysis of differential expression as well as the gene expression levels themselves. The significance of differential behavior, is captured by the *p*-values from a differential expression analysis. These values are transformed into their corresponding *z*-scores, whereby a larger *z*-score denotes a higher significance that the respective gene is differentially expressed. To capture the gene-activation state we determine a gene-specific threshold. To this end, we perform bimodal distribution analysis for each gene over the available conditions and all time-points. Due to the transient (de)activation upon perturbation, genes often show a bimodal distribution in expression values [20], indicating an active and an inactive state. For a gene whose distribution of expression values is a poor match to the bimodal distribution, we use the expression median as a threshold (*cf.* Additional file 1). Based on these values, we define the weight *w* of a gene as follows:

(1) $$w=I\cdot z+\frac{\xi -\vartheta }{\vartheta },$$

where *z* denotes the *z*-score, *ξ *is the expression value, and *ϑ *is the determined threshold value. The trivalued indicator *I* takes values of 1, -1, or 0 if the gene is differentially up- or down-regulated, or shows no differential behavior, respectively. The first term in Equation (1) refers to the differential expression, while the second one combines the normalized difference between the expression value and the gene-specific threshold.

We determine weights for transcriptomics data from the cold and heat stress and the control conditions spanning seven time points (0 - 90 min) and map them onto the genome-scale metabolic network reconstruction of *E. coli* K-12 [21]. If a reaction is annotated with several genes, the AND rule, which accounts for protein complexes, is replaced by using the lowest weight. Moreover, we use the sum of weights if the genes encode isoenzymes and are connected by the OR rule [22]. With this setting, 81% of the reactions in the network can be weighted by experimental data (*cf.* Additional file 1). Furthermore, annotated genes, for which the corresponding gene data are missing, are assigned the median weight of all annotated genes. Reactions that are not associated to a gene in the used network are assigned the median weight over all annotated reactions.

### Data-driven network reduction—the min-max problem

In the following, we develop a formulation of the problem whose solution yields the minimal network of largest weight, quantifying the compliance with the data. More formally, we determine the minimal number of reactions that maximize $\overset{N}{\underset{j=1}{\sum }}w_{j}\cdot v_{j}$, where *w*_*j*_ and *v*_*j*_ are the weight and the flux of reaction *j*, respectively. The problem can be cast as a bilevel mixed-integer linear program (MILP), Equations (2)-(8), where each reaction *j* is assigned a Boolean variable *y*_*j *_∈ {0,1}. If *y*_*j *_= 0, reaction *j* does not carry any flux and is not included in the network; if *y*_*j *_= 1, the reaction carries flux in the range determined by the flux boundaries (Equation (6)). The outer optimization level seeks to minimize the number of reactions in the network (Equation (2)), while fulfilling the inner constraints. The inner problem definition is a modification of the standard flux balance analysis (FBA) [23] formulation (*cf.* Equation (4) and Additional file 1). However, instead of biomass production, the objective function maximizes the sum over weighted fluxes (Equation (3)).

A further constraint on the fluxes is imposed by demanding that a fraction *f*_min_ of the maximum biomass production *f*_max _of the complete network is achieved (Equation (5) and [24]). This requirement is based on optical density data from the same experimental setup, which indicate a growth rate of about 10 to 30% of the growth rate under ambient conditions (*cf.* Additional file 1). To account for the medium (modified MOPS minimal medium) on which the cell cultures were grown, constraints on the exchange reactions are taken from [21] and only inorganic compounds and glucose are allowed to enter and exit the system.

To reduce the computational complexity, we seek to reduce the number of integer variables. To this end, we distinguish between *indispensable* reactions, which make up most of the biomass production and *dispensable* reactions, which have a negligible contributions to growth. To define these two groups, we delete, one by one, every reaction and performed FBA on the perturbed network. If the resulting biomass production remains above a defined threshold (99%), we consider the reaction dispensable for the organism’s viability under ambient conditions (for robustness of the findings, see the Results section). Reactions that are considered indispensable are not assigned a Boolean variable. Altogether, we obtain the following formulation

$$\begin{array}{lll} & \;\text{minimize}\;\overset{N}{\underset{j=1}{\sum }}y_{j}\; & \;\\ & \;\;\;\;y_{j}\; & \;\\ & \;\text{subject to}\;\text{maximize}\;\overset{N}{\underset{j=1}{\sum }}w_{j}\cdot v_{j}\;\text{(Inner)}\; & \;\\ & \;\;\;\;\;\;\;v_{j}\; & \;\\ & \;\;\;\;\left[\begin{array}{ll} \text{subject to} & \overset{N}{\underset{j=1}{\sum }}S_{\text{ij}}\cdot v_{j}=0\\ & \overset{N}{\underset{j=1}{\sum }}c_{j}\cdot v_{i}\geq f_{\text{min}}\\ & 0\leq v_{j}\leq v_{j}^{\text{max}}\cdot y_{j},\forall j\in D\\ & 0\leq v_{j}\leq v_{j}^{\text{max}},\forall j\in N \end{array}\right]\\ & \;\;\;\;y_{j}=\{0,1\},\;\forall j\in D\;\;\;(\text{Outer}),\; & \; \end{array}$$

where *S*_*ij*_ is the stoichiometric coefficient of metabolite *i* in reaction *j*, *c*_*j *_is the contribution of *v*_*j*_ to the objective function and $v_{j}^{\text{max}}$ is the upper boundary on *v*_*j*_, while D and N denote the reactions that are dispensable and indispensable, respectively.

Although we investigate time-series data, the program formulation employs a the quasi-steady-state assumption (Equation (4)). We assume a separation of the time-constants at which transcriptional and metabolic regulations take place. This is justified by the evidence that changes taking place on the metabolic level are generally much faster (seconds) compared to those taking place on the transcriptional level (minutes) [25]. In other words, enzyme dynamics occur more quickly compared to changes in gene expression.

To solve the min-max MILP, it is transformed from a bi-level to a single-level MILP. This procedure employs two steps: (1) finding the dual for the inner linear program [26] and (2) removing the occurring bi-linear terms [27] (*cf.* Additional file 1).

### Fractional appearance of reactions in EFMs

The reduced size of the networks allows the computation of sets of EFMs for the time- and condition specific minimal networks. It has already been shown that the importance of a reaction for network functionality can be characterized by the number of EFMs in which it is involved [13]. Extending this concept to the time domain, we define the fractional appearance *X*_*ij*_ of a reaction *i* at time *j* as the ratio between the number of elementary modes involving reaction *i* and the total number of elementary modes at time *j*: $X(i,j)=\frac{\text{Nr. of EFMs including reaction }\;i\;\text{at time}\;j}{\text{Nr. of all EFMs at time }j}$. This definition allows to characterize the temporal changes in network functionality. A large fractional appearance of a reaction does not only indicate its increased utilization, but also the activation of related processes, which result in an increased contribution of this reaction to the overall number of EFMs.

## Results

### Time- and condition-specific minimal operating networks

For both, cold and heat stress, the minimal networks include 416 to 427 metabolites interconnected by 480 to 486 reactions. The biomass production, as a result of the constraint from the minimization approach, ranges between 0*.*98·10^−5^ and $4.99\cdot 10^{-5}\frac{\mathrm{mol}}{g\cdot \text{DW}-\text{hr}}$.

### Comparison to networks extracted from MADE

We compare the sets of reactions included in the time- and condition-specific networks extracted by both AdaM and MADE. We find that on average 66.6% of reactions are shared between the extracted networks over all time points and conditions, with larger average overlap for the heat condition of 67.2% (percentages with respect to the smaller network, *i.e.*, the network extracted by AdaM). In addition, we determine the expected value of the overlap between the networks extracted by MADE and a random set of reactions of the same size as the networks extracted from our approach. By considering 1000 repetitions, we find this value to be 52.3%, thus confirming the similarity between the two approaches. The results are summarized in Tables 1 and 2.

**Table 1:** Comparison of the network properties for the time- and condition-dependent minimal networks for AdaM and MADE

	**Metabolites**	**Reactions**	**EFMs**
AdaM	416-427	480-486	1060 - 9582
MADE	545	658-806	†
original network	761	1075	†

Given are the number of metabolites and reactions for the networks extracted by our approach and by MADE compared to the original network.

*‡*Note that, with the currently available tools, only our approach extracts minimal networks for which the full set of EFMs can be computed.

**Table 2:** Comparison of the network overlap for the time- and condition-dependent minimal networks between AdaM and MADE

**Time in min**	**Cold**	**Heat**	**Random**
10	62.8	62.6	
20	66.7	68.5	
30	67.8	69.8	
40	63.8	68.3	
50	62.0	67.9	
60	69.0	63.2	
70	70.6	70.2	
average	66.1	67.2	52.3

Given is the overlap in % of reactions included in the networks extracted by our approach and by MADE with respect to the smaller network. The average value for all time-dependent cold- and heat-shock specific networks are significantly higher than the values for a randomly drawn set of reactions of the same size.

### EFM-based characterization of adaptability

The number of EFMs in the minimal networks ranges between 1060 and 9582, which is small compared to other metabolic network models of similar size [13,18]. The extracted networks include only putatively active reactions and no inactive alternative pathways, and thus capture a specific metabolic state of low flexibility. In the following, the sets of EFMs are analyzed with respect to: (1) robustness to variations in the network extraction process, (2) stress adaptation of the network as a whole, and (3) the transitional behavior of individual reaction.

#### Robustness of the optimization approach

We investigate the robustness of the approach with respect to: (1) threshold variations for the optimization approach and (2) slightly suboptimal networks. To address the first aspect, we repeat the network minimization for three different thresholds to distinguish between dispensable and indispensable reactions. In addition, we examine three different values of minimal biomass production (*i.e.*, 1%, 10% and 20% of the optimal biomass in the original network). To compare the results, we use the intersection of reactions in the minimal networks for each time point. The average overlap of reactions is 94.4% for cold and 94.3% for heat (with respect to the smaller network) when comparing all possible parameter sets. When comparing the average overlap of networks for different time-points, we find it to be 94.1% for both cold and heat shock, indicating bigger similarity for networks extracted with different thresholds than for different time points.

To test the behavior of slightly suboptimal networks, we add noise to the weights of 100 randomly selected reactions. As a maximum noise level, we set 1% of the total range of weights. We repeat the analysis for different time points and threshold values, resulting in more than 1000 network perturbations. Comparing the resulting sets of EFMs, we find an average overlap of 82.0% over pairs of EFM sets. Therefore, the considered network perturbations together with variations in the used threshold values confirm the robustness of the extracted networks with respect to EFMs. Furthermore, this suggests that EFMs can be used to develop time-resolved descriptors of reactions’ contribution to network functionality.

#### Cold and heat stress response show distinct temporal behavior

**Figure 2:**
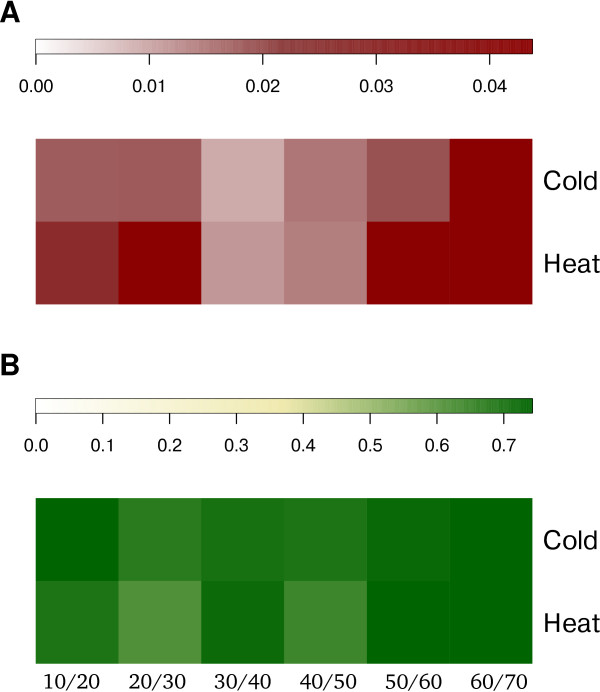
**Global characterization of temporal adaptation-related networks.** Transitional behavior of the metabolic network after heat and cold stress perturbation. Heatmap of the Jaccard index from **(A)** sets of EFMs and **(B)** sets of dispensable reactions included in the minimal networks from consecutive time-points. A low Jaccard index denotes dissimilarity.

To investigate the global properties of the transition, we determine the similarity for consecutive sets of EFMs and sets of dispensable reactions by using the Jaccard index. Changes in the usage of EFMs as well as dispensable reactions over time suggest adaptation-relation processes. The results are illustrated in Figure 2, showing heatmaps of the Jaccard index for cold and heat shock.

The values for the Jaccard index for consecutive sets of EFMs for the minimal cold stress networks are slightly lower (0.009-0.252) compared to those from the heat stress networks (0.012-0.271). These changes in the usage of EFMs, resulting from data-driven network extraction, can be regarded as changes in the distribution of fluxes through the networks. Moreover, the values for the Jaccard index for dispensable reactions remaining in the minimal networks are similar for cold (0.697-0.759) and heat stress (0.618-0.771), suggesting similar changes in activation patterns of reactions.

Considering the patterns of change, for cold shock we observe the first strong dissimilarity between sets of EFMs between 0 and 20 min after stress application, indicating that the main metabolic response takes place in this time range. For heat shock, the onset of the response is slightly shifted in time. These findings suggest that the stress response for the two conditions takes place on two slightly different time regimes. Such tendency can also be observed when comparing the time course of the similarities for the sets of dispensable reactions. In addition, for both conditions, the peak in dissimilarity between sets of EFMs occurs between 30 and 50 minutes. Between 50 and 90 minutes, both condition-specific networks exhibit similarity with respect to their sets of EFMs and sets of dispensable reactions, demonstrating that the system has started settling in the new condition.

### Fractional appearance of reactions in EFMs discriminates two types of reactions

For ease of interpretation, we focus our analysis on the 50 reactions of highest fractional appearance for each time-point. The union of these selected reactions over all time points contains 71 and 76 reactions for the cold and heat shock, respectively. Out of these, 43 are conserved between the two stresses.

To gain general insights into the patterns of the fractional appearance profiles of these selected reactions, we determined the Kendall correlation *τ*(*cf.* Additional file 1). The value captures (dis)similarities in the temporal usage between reactions. A heatmap representation of the correlation matrix is shown in the Additional file 1: Figure S1. The results hint at the presence of different temporal patterns apparent from the clustering.

The working hypothesis is twofold: Reactions that are grouped together are expected to belong to the same pathways, or are regulated in a similar manner (*e.g.*, by the same allosteric regulator). Furthermore, groups of reactions exhibiting mostly negative correlation towards other reactions indicate different patterns of usage over time and are probably stress induced.

**Figure 3Figure 3:**
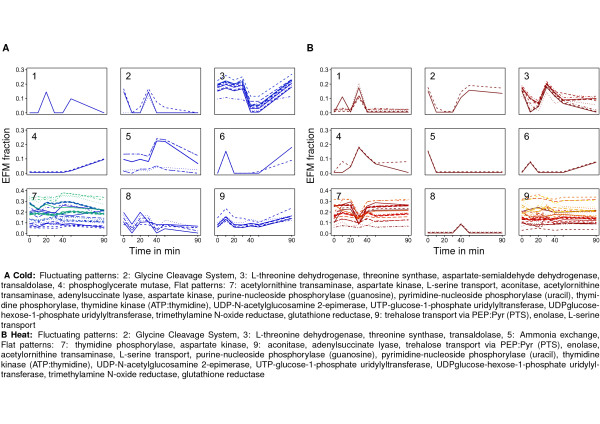
**Clustering of time-resolved fractional appearance profiles.** Shown are the fractional appearance profiles of reactions over time. Under both **(A)** cold and **(B)** heat shock, the selected reactions group into 9 clusters. Enzyme names discussed in the text are given. A complete list of all enzyme names is given in the Additional file 1: Table S3. In both stresses clusters 7 and 9 represent metabolic reactions that are constantly active, most of them crucial for viability. All other clusters represent reactions that undergo (de)activation and are likely involved in stress response. Note: For better visualization, identical profiles are slightly shifted. Reference value is given by the profile appearing on the bottom.

To further investigate this hypothesis we cluster the fractional appearance profiles of the previously selected reactions (*cf.* Materials and Methods). The results of the clustering are shown in Figure 3 (a full list of all clustered reaction names can be found in the Additional file 1: Table S2). The shape of the profile in each cluster suggests two groups of reactions: those which are active across all time points, represented by flat profiles, and those whose usage changes during the progression of stress application, exhibiting fluctuating patterns. More specifically, a reaction which exhibits fractional appearance greater than zero over all time points is considered to have a flat profile. In contrast, a reaction which exhibits fractional appearance of zero in at least one time point is considered to have a fluctuating pattern. Depending on the employed clustering method, there could be clusters exclusively composed of reactions showing flat or fluctuating profiles as well as clusters containing reactions of both profiles. For instance, for the clustering of fractional appearance profiles under cold stress in Figure 3, we observe that clusters 1, 2, 4, and 6 consist only of reactions with fluctuating profiles, clusters 7 and 9 of reactions with flat profiles, while clusters 3, 5, and 8 include reactions of both types of profiles.

#### Flat patterns represent indispensable metabolic reactions

We first focus our analysis on reactions whose occurrence in EFMs does not change as a result of perturbation, *i.e.*, on clusters in which all fractional appearance profiles are flat. Those reactions are grouped in cluster 7 and 9 in both cold and heat stress (Figure 3).

In total, these clusters consist of 36 and 47 reactions for cold and heat stress, respectively, of which 19 of these reactions are conserved between the two stresses. These reactions appear in 5 to 40% of all EFMs, which indicates a major role for network functionality. To gain a general overview of the higher biological processes of this set of reactions, we perform GO term over-representation analysis on their annotated genes (Additional file 1: Table S3). The clusters show significant enrichment for many processes crucial for cell growth, including amino acids (*e.g.*, homoserine, serine, methionine, and lysine) biosynthesis, nucleotide, nucleoside and nucleobase interconversions, amine biosynthesis, coenzyme and carboxylic acid metabolic processes as well as tricarboxylic acid (TCA) cycle. The reactions can further be grouped according to specific pathways.

Closer inspection reveals a group of 3 constantly used reactions - aconitase, trehalose transport via PEP:Pyr (PTS) and enolase, which belong to the central carbon metabolism. The constant utilization of glucose uptake system (PTS) is not surprising, since it is crucial for culture grown on this nutrient, as well as for the formation of G6P and flux from PEP to pyruvate. Furthermore, this reaction has also been shown to be constitutively active under different nutritional perturbations in *E. coli*[28].

The second group contains reactions involved in amino acid metabolism, a process crucial for protein synthesis. Those reactions include acetylornithine transaminase, adenylsuccinate lyase, aspartate kinase, and L-serine transport. Another group contains reactions involved in nucleotides biosynthesis and degradation, processes essential for transcription and replication, including: purine-nucleoside phosphorylase (guanosine), pyrimidine-nucleoside phosphorylase (uracil), and thymidine phosphorylase, as well as thymidine kinase (ATP:thymidine). This group extends to three reactions involved in amino- and nucleotide-sugar metabolism, namely, UDP-N-acetylglucosamine 2-epimerase, UTP-glucose-1-phosphate uridylyltransferase, and UDP-glucose-hexose-1-phosphate uridylyltransferase.

Finally, we observe constant utilization of glutathione reductase and trimethylamine N-oxide reductase. The first can be understood by the fact that the ratio of reduced to oxidized glutathione in *E. coli* is kept on a high ratio, ensuring proper maintenance of reduced thiol groups, protection against oxidative damage, and formation of deoxyribonucleotide precursors for DNA synthesis [29]. The second is a part of the electron transport chain.

The results of the functional enrichment analysis and biological interpretation of the metabolic role of indispensable reactions, showing flat profiles of fractional appearance, supports our assumption that these reactions constitute the most crucial part of the metabolic network.

#### Fluctuating patterns capture condition-specific temporal response

Next we investigate reactions whose temporal appearance in EFMs changes as a result of the applied stress. The number of reactions showing such behavior is smaller compared to that of reactions which are constantly used. There are 35 reactions for cold stress and 29 for heat stress.

Over-representation analysis of biological processes reveals that under cold stress cluster 2 is enriched for catabolic processes, in particular, of amino acids, organic acids, and coenzymes, followed by acetyl-CoA biosynthetic process from pyruvate, and glycolysis. The reactions in this cluster are excluded from the networks at 10 min and peak with respect to their fractional appearances at 30 min. A prominent representative in this group of reactions is the glycine cleavage system, which has been found to be slightly affected by cold stress [30].

Cluster 3 is enriched for biosynthesis of the aspartate family of amino acids (*i.e.*, homoserine and threonine), carbohydrate (glucose) catabolic process, and pentose-phosphate shunt, among other more general GO terms. The fractional appearance profiles for the included reactions remain unchanged up to 30 min, after which the reactions are excluded from the time-specific networks to be reintroduced 50 min after application of the stress. This cluster includes the reactions: L-threonine dehydrogenase, threonine synthase, and aspartate-semialdehyde dehydrogenase. This suggest that even some prominent pathways, such as amino acid synthesis, are not constantly kept at high level throughout adaptation to the stress. Moreover, transaldolase appears to undergo the same transition in fractional appearance, which is due to the observation that, unlike in exponential growth, cells facing cold stress transiently use this reaction to convert two molecules of fructose-6-phosphate and one molecule of 3PGA [19,31].

The coupling between the pentose phosphate pathway and catabolic processes is also apparent in the enrichment of GO terms in cluster 4. Here, the two considered reactions are only present in the extracted networks for the last time points. One of these reactions, phosphoglycerate mutase, takes part in glycolysis, which together with glucose consumption is reduced under low temperatures, especially in the early time points after stress [30].

Under heat stress, cluster 2 consists of profiles where the reactions are initially used, then excluded from the network, and finally reintroduced. Over-representation analysis demonstrates that catabolic processes involving amino acids, glyoxylate and coenzymes are enriched. Interestingly, the reactions in this cluster are also grouped together in cluster 2 under cold stress. However, it appears that after initial usage of the glycine cleavage system under heat stress, it is transiently shut down in a manner opposite of that under cold stress.

Cluster 5 includes ammonium exchange which is down-regulated after application of heat stress. This is in line with the catabolic processes observed in cluster 2, suggesting that protein synthesis is present to support maintenance of cell vitality without the need to sustain growth. In addition, cluster 3 under heat stress has a high overlap with cluster 3 under cold stress. However, the patterns of fractional appearance, as already observed for cluster 2, show a different temporal behavior. We therefore suggest the hypothesis that although same biological processes are involved in adaptation to temperature stresses, the temporal usage in terms of (in)activation may slightly differ. The activation pattern of these processes may further amplify the effect of genes specific to cold/heat stress.

## Discussion

Here we proposed a novel approach to investigate adaptation of metabolism upon external perturbation. Based on experimental data we determine time- and condition-specific minimal networks for which sets of EFMs can be calculated. These sets are used to determine the fractional appearance profiles of reactions. This integrative profile combines information from transcriptomics data, the underlying network structure, and biologically meaningful flux distributions in a quasi steady-state; thus it includes information which transcriptomics data would never be able to reveal on their own.

The fractional appearance of reactions has already been investigated with respect to the concept of robustness [13]. Here we demonstrate that expanding this concept to the time domain facilitates the distinction of two types of patterns—flat and fluctuating. In light of the differences as well as the overlap between the concepts of robustness and adaptability, the reactions exhibiting fluctuating patterns are the first candidates that drive the adaptation of the system upon perturbation. Moreover, like transcript data, the fractional appearance profiles can also be subjected to clustering and enrichment analyses (with respect to a chosen ontology). With the help of these analyses, our approach allows the identification of adaptation-related processes.

It must be noted that our proposed approach extracts network for individual time points, without accounting for their dependency in the time domain. However, since the weighting of the reactions is conducted by using data which already embed the temporal dependency, this also extends to the extracted networks.

Since transcriptomics data do not necessarily reflect enzyme activities (due to post-transcriptional modification and regulatory effects), we use the results from the analysis of the expression data only as indicator for the activity of the respective reaction rather that definite values. Furthermore, the approach does not rely on a condition-specific objective function, *e.g.*, biomass yield. Thus, it overcomes one of the drawbacks of FBA, associated with the selection of a suitable objective function, not only for different but also varying conditions. Finally, comparative analysis with MADE, a state-of-the-art method, demonstrates high overlap between the extracted networks. However, in comparison to MADE, our approach results in consistently smaller networks amenable to EFM analysis.

## Conclusion

We applied our approach to time-resolved transcriptomics data from heat and cold shock experiments in *E. coli*. The predictions from the integration of the large-scale metabolic networks with time-series data are in line with observations and conclusions from existing experimental studies. Moreover, analysis of the fractional appearance profiles for heat and cold stress adaptation in *E. coli* have generated interesting hypothesis to be validated in future experiments. Finally, the proposed method and the presence of the two types of profiles, resulting from its application on a well-investigated model organism, indicate the “tug-of-war” between the systemic properties of robustness and adaptability necessary for maintenance of major processes while settling in a new metabolic state.

## Competing interests

The authors declare that they have no competing interests.

## Authors’ contributions

NT and ZN designed the study and wrote the manuscript. NT performed the study. SJ, NT and ZN interpreted the results. All authors read and approved the final manuscript.

## Supplementary Material

Additional file 1 Supplementary Information. Click here for file
