# Supplementary material for: Integration of time-resolved transcriptomics data with flux-based methods reveals stress-induced metabolic adaptation in Escherichia coli
Source: BMC Syst Biol. 2012 Nov 30;6:148. doi: 10.1186/1752-0509-6-148 (PMC3576321; doi:10.1186/1752-0509-6-148)
Supplement: Additional file 1 — Supplementary Information. [file 1752-0509-6-148-S1.pdf]

Supplementary Information: Integration of time-resolved transcriptomics data with flux-based methods reveals stress-induced metabolic adaptation in *Escherichia coli*

Nadine Töpfer[1], Szymon Jozefczuk[2] and Zoran Nikoloski*[1]

[1]Systems Biology and Mathematical Modeling Group, Max-Planck-Institute of Molecular Plant Physiology, 14476 Potsdam, Germany
[2] ETH Zurich, Institute of Molecular Systems Biology, 8093 Zurich, Switzerland

Table of Contents

Supplementary Methods

1 Experimental Methods **2**

2 Theoretical Methods **2**
2.1 Weighting of reactions 2
2.2 Flux-based methods 2
2.2.1 Flux balance analysis 3
2.2.2 Elementary flux modes 3
2.3 Finding the dual problem 3
2.4 Kendall rank correlation coefficient 6
2.5 Clustering analysis 6
2.6 GO enrichment analysis 7

Supplementary Tables

S1 Approximated growth rates from optical density measurements 8
S2 Clustering of selected reactions by their fractional appearance profiles 9
S3 Summary of overrepresented GO Terms 11

Supplementary Figures

S1 Heatmaps of the Kendall correlation for fractional appearance profiles. 16
S2 Histogram of gene expression values 17
S3 Distributions of time- and condition-specific weights 17

*Nikoloski@mpimp-golm.mpg.de

1 Experimental Methods

A detailed description of the experimental procedure, the transcript data extraction and normalization can be found in the Materials and Methods section of [1]. To approximate the growth rate of the cells under stress condition, we assume that the change in optical density (OD) scales linearly with the biomass production of the cells. To avoid the introduction of additional uncertainties, we also make the following simplifying assumption: OD-specific cell concentrations do not differ significantly for different growth conditions and the biomass production is approximately the same after both heat and cold shock. From the OD data of the supplementary file [1], we calculate the mean OD values of the tree replicates for each time point and determine the differences between time intervals for the control, cold and heat condition. Based on these values, we approximate the growth rate with respect to the control condition. The results are summarized in Table S1. We consider a biomass production of the minimized network of at least 10% of the original network as a valid optimization criterion.

2 Theoretical Methods

2.1 Weighting of reactions

Statistical analysis of the gene expression data is performed using the software environment R [2] and the packages "limma" [3] (for differential gene-expression analysis) and "mixtools" [4] (for bimodal distribution analysis). The data are log-normalized and differential gene expression is determined for each time-point between the replicates of the control and the respective stress condition. The expression values of gene i for all time-points and conditions (including also data from lactose shift and oxidative stress experiments for five time-points) are used as an approximation of the whole spectrum of possible expression values. As a threshold for calling a distribution bimodal or not, the median of all log-likelihoods of the fitted curves (*normalmix*) is used. For those genes showing a bimodal distribution, the intersection of both fitted curves determines the gene-specific threshold value ϑ. If the gene shows no bimodality, the median of all expression values for the respective gene is used as a threshold (for an example see Fig. S1).

We use the genome-scale metabolic network reconstruction of *E. coli* K-12 [5]. The network contains 153 external metabolites. To avoid that they become dead-end metabolites, i.e., are only consumed or produced, they are removed form the network. Out of the 1075 reactions of the network, 871 can be weighted by experimental data. After splitting reversible reactions into a forward and a backward reaction the resulting network consists of 618 metabolites and 1331 reactions. Two example distributions of weights mapped onto the network are shown in Figure S2.

2.2 Flux-based methods

Flux-based methods employ the stoichiometry of a metabolic network and constraints on the reaction velocities (*e.g.*, upper and lower boundaries, reaction reversibility) to predict steady-state flux distributions. These methods allow the investigation of biochemical network, even when detailed kinetics of the reactions are unknown or uncertain, which is the case for most biochemical networks. The stoichiometry of all reactions of the network is represented by a matrix S, where S_{ij} denotes the stoichiometric coefficient of metabolite i in reaction j. For a system of n metabolites and m reactions S is an n x m matrix. Depending on the metabolic state, each reaction j proceeds at a certain reaction velocity v_j. At steady-state the system is mass-balanced and constrained to fulfill the following equation:

$$S \cdot v = 0 \ ,$$

where v is the vector of fluxes. Further constraints on the system are imposed by assigning boundaries to the reaction velocities:

$$v^{\min} \leqslant v \leqslant v^{\max} \ ,$$

where $v^{\min}$ and $v^{\max}$ are the upper and lower boundaries, respectively. These constraints result from the availability of nutrients in the growth media, maximal enzyme activities or thermodynamic constraints. If a reaction v_j is irreversible, the corresponding lower boundary $v_j^{\min}$ is zero. For most biological networks there are more reactions than metabolites and therefore the system is under-determined [6]. This implies that the imposed constraints do not define a unique solution, but rather a solution space Φ, which is a convex set in the m-dimensional space of fluxes.

2.2.1 Flux balance analysis

Flux balance analysis (FBA) is a flux-based method that aims at optimizing a certain objective function. For metabolic models of microorganisms the maximization of growth has been shown a valid optimization goal [7, 8]. This optimality condition can be combined with the steady-state constraints by introducing a biomass reaction with flux v_{bm}. This reaction produces biomass from a linear combination of several metabolic precursors in the network. The resulting mathematical formulation is the following:

$$\begin{array}{rl} \text{maximize} & c \cdot v \\ \text{subject to} & S \cdot v = 0 \\ & v^{\min} \leqslant v \leqslant v^{\max} \ , \end{array}$$

where c is a vector of weights, indicating how much each reaction contributes to the objective function. In the case when only the biomass production is optimized, c is a vector of zeros with only one entry equal to one at the position of v_{bm}. By using this formulation, one can determine points within Φ, that describe optimal flux distributions with respect to the objective function. Due to its linear formulation FBA is readily applicable to large-scale networks.

2.2.2 Elementary flux modes

An elementary flux mode (EFM) is defined as a minimal set of enzymes that can operate at steady-state, with all irreversible reactions involved used in the appropriate direction [9]. In contrast to FBA, EFM-based analysis does not predict optimal flux distributions, as it captures the whole set of non-decomposable fluxes. Therefore, any possible flux distribution of the metabolic system can be represented as a linear combination of its EFMs.

2.3 Finding the dual problem

The fist step in transforming a bi-level to a single-level mixed-integer linear optimization problem (MILP) is to find the dual for the inner linear program. From duality theory [10], it follows that for each linear problem that can be rewritten in the standard maximum problem form:

$$\begin{array}{rl} \text{maximize} & c \cdot x \\ \text{subject to} & A \cdot x \leqslant b \\ & x \geqslant 0 \ , \end{array}$$

there is a dual standard minimum form:

$$\begin{array}{rl} \text{minimize} & \mu^T \cdot b \\ \text{subject to} & \mu^T \cdot A \geqslant c^T \\ & \mu \geqslant 0 \ , \end{array}$$

where x and μ represent the vector of variables of the dual and the primal, respectively, c and b are vectors of coefficients and A is a matrix of coefficients. From the strong duality theorem [10], it follows that, if the primal has an optimal solution x^* and the dual also has an optimal solution μ^*, their objective function values must be equal :

$$c \cdot x^* = \mu^{*T} \cdot b \ . \tag{1}$$

Our bilevel program is the following:

$$\begin{array}{lll}
\underset{y_j}{\text{minimize}} & \sum_{j=1}^{N} y_j & \\
\text{subject to} & \underset{v_j}{\text{maximize}} \quad \sum_{j=1}^{N} w_j \cdot v_j & \text{(Inner)} \\
& \left[\begin{array}{ll}
\text{subject to} & \sum_{j=1}^{N} S_{ij} \cdot v_j = 0 \\
& \sum_{j=1}^{N} c_j \cdot v_i \geqslant f_{\min} \\
& 0 \leqslant v_j \leqslant v_j^{\max} \cdot y_j, \quad \forall j \in \mathcal{D} \\
& 0 \leqslant v_j \leqslant v_j^{\max}, \quad \forall j \in \mathcal{N}
\end{array} \right] & \\
& y_j = \{0,1\}, \quad \forall j \in \mathcal{D} & \text{(Outer)} \ ,
\end{array}$$

where y_j is the Boolean variable and w_j the weight associated with reaction j, c_j is the contribution of v_j to the biomass production, f_{min} is a requirement for a minimal biomass production and $v_j^{\max}$ is the upper boundary on v_j, while $\mathcal{D}$, $\mathcal{N}$ denote the set of dispensable and nondispensable reactions, respectively. We use that equality condition Eq. (1) to transform the inner optimization problem into a non-linear system of equality constraints.

This transformation leads to a single-level mixed-integer problem:

$$
\begin{aligned}
\min \quad & \sum_{j=1}^{N} y_j \\
\text{s.t.} \quad & \mu_{bio} \cdot f_{\min} + \sum_{j \in \mathcal{D}} \mu_j^{\max} \cdot v_j^{\max} \cdot y_j + \sum_{j \in \mathcal{N}} \mu_j^{\max} \cdot v_j^{\max} = \sum_{j=1}^{N} w_i \cdot v_i \\
& \sum_{j=1}^{N} S_{ij} \cdot v_j = 0 \\
& \sum_{j=1}^{N} c_j \cdot v_i \geqslant f_{\min} \\
& \sum_{i=1}^{N} \mu_i \cdot S_{ij} + \mu_{bio} \cdot c_j + \mu_j^{\max} \geqslant w_j, \qquad \forall j \in \mathcal{D} \\
& v_j \leqslant v_j^{\max} \cdot y_j, \qquad \forall j \in \mathcal{D} \\
& v_j \leqslant v_j^{\max}, \qquad \forall j \in \mathcal{N} \\
& \mu_{\max} \geqslant 0 \\
& \mu_i, \mu_{bio}, \qquad \text{unrestricted} \\
& y_j = \{0, 1\}, \qquad \forall j \in \mathcal{D}\ ,
\end{aligned}
$$

where μ_i, μ_{bio}, $\mu_i^{\max}$ are the dual variables associated with the coefficients of the stoichiometric matrix S_{ij}, the biomass function and the upper boundaries for the reaction velocities v_j^{max}, respectively.

By introducing new auxiliary variables [11], the non-linearities now occurring in the constraints, can be recast into equivalent linear expressions. For each non-linearity we introduce two new continuous variables $q^{\max}$ and $r^{\max}$, where $q_j^{\max} = \mu_j^{\max} \cdot y_j$. The continuous variable $r^{\max}$ satisfies:

$$
q_j^{\max} = \mu_j^{\max} - r_j^{\max}\ .
$$

We introduce new inequalities

$$
\begin{aligned}
\mu_{j,low}^{\max} \cdot y_j \leqslant q_j^{\max} \leqslant \mu_{j,up}^{\max} \cdot y_j \\
\mu_{j,low}^{\max} \cdot (1 - y_j) \leqslant r_j^{\max} \leqslant \mu_{j,up}^{\max} \cdot (1 - y_j)\ ,
\end{aligned}
$$

where $\mu_{j,low}^{\max}$ and $\mu_{j,up}^{\max}$ are the lower and upper boundaries for $\mu_{j,l}^{\max}$, respectively. They can be calculated by minimizing and maximizing the value of $\mu_{j,low}^{\max}$ and $\mu_{j,up}^{\max}$ subject to the constraints of the dual problem, respectively [12].

The bi-level problem definition is now transformed into the following MILP:

$$
\begin{aligned}
\min \quad & \sum_{j=1}^{N} y_j \\
\text{s.t.} \quad & \mu_{bio} \cdot f_{\min} + \sum_{j \in \mathcal{D}} v_j^{\max} \cdot q_j^{\max} + \sum_{j \in \mathcal{N}} \mu_j^{\max} \cdot v_j^{\max} = \sum_{j=1}^{N} w_j \cdot v_j \\
& \sum_{j=1}^{N} S_{ij} \cdot v_j = 0 \\
& \sum_{j=1}^{N} c_j \cdot v_j \geqslant f_{\min} \\
& \sum_{i=1}^{N} \mu_i \cdot S_{ij} + \mu_{bio} \cdot c_j + \mu_j^{\max} \geqslant w_j, \qquad \forall j \in \mathcal{D} \\
& v_j \leqslant v_j^{\max} \cdot y_j, \qquad \forall j \in \mathcal{D} \\
& v_j \leqslant v_j^{\max}, \qquad \forall j \in \mathcal{N} \\
& q_j^{\max} = \mu_j^{\max} - r_j^{\max}, \qquad \forall j \in \mathcal{D} \\
& \mu_{j,low}^{\max} \cdot y_j \leqslant q_j^{\max} \leqslant \mu_{j,up}^{\max} \cdot y_j, \qquad \forall j \in \mathcal{D} \\
& \mu_{j,low}^{\max} \cdot (1 - y_j) \leqslant r_j^{\max} \leqslant \mu_{j,up}^{\max} \cdot (1 - y_j), \qquad \forall j \in \mathcal{D} \\
& \mu_j^{\max} \geqslant 0, \qquad \forall j \in \mathcal{D} \\
& \mu_i, \mu_{bio} \qquad \text{unrestricted} \\
& y_j = \{0, 1\}, \qquad \forall j \in \mathcal{D}\ ,
\end{aligned}
$$

which is implemented and solved for different set of weights.

The metabolic model of *E. coli* K-12 [5] is imported into Matlab [13] using the SBMLToolbox [14]. The optimization approaches (the network minimization program and FBA) are implemented in Matlab using Tomlab 7.8 [15]. EFMs in the time- and condition-specific minimal networks are computed using efmtool [16].

2.4 Kendall rank correlation coefficient

Let $a = (a_1, ...a_n)$ and $b = (b_1, ...b_n)$ be two sets of observations from two variables A and B respectively, such that all the values of a_i and b_i are unique. Any pair of observations (a_i, b_i) and (a_j, b_j) are concordant if both $a_i > a_j$ and $b_i > b_j$ or if both $a_i < a_j$ and $b_i < b_j$. If $a_i > a_j$ and $b_i < b_j$ or if $a_i < a_j$ and $b_i > b_j$, they are disconcordant. The Kendall coefficient is defined as: $\tau = \frac{(\text{number of concordant pairs})-(\text{number of discordant pairs})}{\frac{1}{2}n(n-1)}$ and takes values between $[-1, 1]$. If the two sets are independent their Kendall τ coefficient is zero. If two rankings match perfectly, the coefficient takes a value of 1 and a value of -1, if the two sets have complete disagreement.

2.5 Clustering analysis

The clustering of the fractional appearance profiles is performed using the package "cluster" [17] and the function "PAM", using Pearson correlation as a distance measure. It is repeated 100 times for each number of clusters k between 2 and 10. The Silhouette index [18] is used to determine the most appropriate number of clusters.

2.6 GO enrichment analysis

To perform GO enrichment analysis for the selected reactions, we use the gene-reaction annotation of the metabolic network reconstruction to backtrack reaction related genes. For these genes, the blattner gene IDs are translated to Entrez IDs. The R package "hyperGTest" [19] and the org.EcK12.eg.db [20] are used to determine significant terms in the biological process ontology, with a significance level of 0.05.

Table S1. Approximated growth rates from optical density measurements. Given are the delta mean OD values (Δ∅OD) of three replicates for consecutive time-points $t_{x/(x+10)}$ and the approximated growth rate with respect to the control condition.

condition	$t_{0/10}$	$t_{10/20}$	$t_{20/30}$	$t_{30/40}$	$t_{40/50}$
Δ∅OD					
control	0.09	0.06	0.06	0.09	0.08
cold	0.03	-0.03	0.02	0.01	0.03
heat	0.05	0.04	0.00	0.02	0.01
% of growth under optimal control conditions					
cold	28.83	-41.03	34.78	14.81	33.33
heat	57.66	56.41	0.00	18.52	12.50

Table S2. Clustering of selected reactions by their fractional appearance profiles for cold and heat shock, respectively. Listed are the enzyme names for the respective reactions for each of the nine clusters. If a reaction is reversible, its proceeding direction is indicated by -f and -r, denoting forward- or backward, respectively.

Cold stress	Heat stress
Cluster 1	Cluster 1
acetolactate synthase	acetolactate synthase<br>adenine transport via proton symport-f<br>NAD synthase (nh3)<br>nucleoside-diphosphate kinase (ATP:dADP)-b<br>phosphoglycerate mutase-b
Cluster 2	Cluster 2
UDP-N-acetyl-D-mannosaminuronic acid transferase<br>Glycine Cleavage System<br>6-hydroxymethyl-dihydropterin pyrophosphokinase	UDP-N-acetyl-D-mannosaminuronic acid transferase<br>Glycine Cleavage System
Cluster 3	Cluster 3
acyl-carrier protein synthase<br>L-Cysteine exchange<br>phosphoribosylpyrophosphate synthetase-f<br>D-ribose transport via ABC system<br>dTDP-4-dehydrorhamnose 3;5-epimerase<br>L-threonine dehydrogenase<br>threonine synthase<br>aspartate-semialdehyde dehydrogenase-b<br>L-idonate transport via proton symport-b<br>nucleoside-diphosphate kinase (ATP:CDP)-b<br>tagaturonate reductase-b<br>transaldolase-b	acyl-carrier protein synthase<br>phosphoribosylpyrophosphate synthetase-f<br>riboflavin synthase<br>D-ribose transport via ABC system<br>dTDP-4-dehydrorhamnose 3;5-epimerase<br>L-threonine dehydrogenase<br>threonine synthase<br>gamma-butyrobetainyl-CoA: carnitine CoA transferase-b<br>L-idonate transport via proton symport-b<br>nucleoside-diphosphate kinase (ATP:CDP)-b<br>ornithine carbamoyltransferase-b<br>tagaturonate reductase-b<br>transaldolase-b
Cluster 4	Cluster 4
adenine transport via proton symport-f<br>phosphoglycerate mutase-b	CO2 exchange<br>spermidine transport via ABC system
Cluster 5	Cluster 5
NMN exchange<br>Phosphatidylserine decarboxylase<br>purine-nucleoside phosphorylase (Inosine)-f<br>D-sorbitol transport via PEP:Pyr PTS<br>thymidylate synthase<br>UDP-N-acetyl-D-mannosamine oxidoreductase<br>adentylate kinase (GTP)-b<br>inosine transport in via proton symport-b<br>Maltodextrin phosphorylase (maltoheptaose)-b	Ammonia exchange<br>aspartate-semialdehyde dehydrogenase-b
Cluster 6	Cluster 6
NAD synthase (nh3)<br>nucleoside-diphosphate kinase (ATP:dADP)-b	L-Cysteine exchange<br>phosphoribosylpyrophosphate synthetase-b
Cluster 7	Cluster 7
purine-nucleoside phosphorylase (Deoxyguanosine)-f<br>pyruvate reversible transport via proton symport-f<br>L-rhamnose isomerase-f<br>rhamnulokinase<br>L-serine reversible transport via proton symport-f<br>Trimethylamine N-oxide reductase (menaquinol 8)<br>thymidine kinase (ATP:thymidine)<br>L-tyrosine reversible transport via proton symport-f<br>UDP-N-acetylglucosamine 2-epimerase<br>Ureidoglycolate hydrolase<br>D-xylose transport in via proton symport<br>aconitase-b<br>acetylornithine transaminase-b<br>adenylsuccinate lyase-b	NMN exchange<br>Phosphatidylserine decarboxylase<br>pyruvate reversible transport via proton symport-f<br>quinolinate synthase<br>L-rhamnose isomerase-f<br>rhamnulokinase<br>D-sorbitol transport via PEP:Pyr PTS<br>thymidine kinase (ATP:thymidine)<br>UDP-N-acetyl-D-mannosamine oxidoreductase<br>aspartate kinase-b<br>dethiobiotin synthase-b<br>2-dehydro-3-deoxy-D-gluconate transport via proton symport-b<br>Dihydroxyacetone transport via facilitated diffusion-b<br>dihydrofolate reductase-b

Continued on next page

Table S2 – continued from previous page	
aspartate kinase-b<br>dethiobiotin synthase-b<br>2-dehydro-3-deoxy-D-gluconate transport via proton symport-b<br>dihydrofolate reductase-b<br>UTP-glucose-1-phosphate uridylyltransferase-b<br>D-glucarate transport via proton symport-b<br>D-glucuronate transport via proton symport-b<br>L-glutamate transport via proton symport-b<br>glutathione oxidoreductase-b<br>orotate phosphoribosyltransferase-b<br>purine-nucleoside phosphorylase (Guanosine)-b<br>pyrimidine-nucleoside phosphorylase (uracil)-b<br>thymidine phosphorylase-b<br>L-tyrosine reversible transport via proton symport-b<br>UDP-glucose-hexose-1-phosphate uridylyltransferase-b	UTP-glucose-1-phosphate uridylyltransferase-b<br>D-glucuronate transport via proton symport-b<br>L-glutamate transport via proton symport-b<br>inosine transport in via proton symport-b<br>thymidine phosphorylase-b<br>L-tyrosine reversible transport via proton symport-b
Cluster 8	Cluster 8
riboflavin synthase<br>S-ribosylhomocysteine cleavage enzyme<br>gamma-butyrobetainyl-CoA: carnitine CoA transferase-b<br>Hypoxanthine transport-b<br>ornithine carbamoyltransferase-b<br>thiamine-phosphate kinase-b	Fructose transport via PEP:Pyr PTS (f6p generating)<br>phosphopantothenate-cysteine ligase<br>uridylate kinase (dUMP)-b
Cluster 9	Cluster 9
L-ribulose-phosphate 4-epimerase-f<br>thymidine transport in via proton symport<br>trehalose transport via PEP:Pyr PTS<br>enolase-b<br>phosphopentomutase-b<br>Rhamnulose-1-phosphate aldolase-b<br>L-serine reversible transport via proton symport-b	purine-nucleoside phosphorylase (Deoxyguanosine)-f<br>purine-nucleoside phosphorylase (Inosine)-f<br>L-ribulose-phosphate 4-epimerase-f<br>UDP-glucose-hexose-1-phosphate uridylyltransferase-b<br>L-serine reversible transport via proton symport-f<br>thymidine transport in via proton symport<br>Trimethylamine N-oxide reductase (menaquinol 8)<br>trehalose transport via PEP:Pyr PTS<br>L-tyrosine reversible transport via proton symport-f<br>UDP-N-acetylglucosamine 2-epimerase<br>Ureidoglycolate hydrolase<br>D-xylose transport in via proton symport<br>aconitase-b<br>acetylornithine transaminase-b<br>adenylsuccinate lyase-b<br>enolase-b<br>glutathione oxidoreductase-b<br>Hypoxanthine transport-b<br>orotate phosphoribosyltransferase-b<br>phosphopentomutase-b<br>purine-nucleoside phosphorylase (Guanosine)-b<br>pyrimidine-nucleoside phosphorylase (uracil)-b<br>Rhamnulose-1-phosphate aldolase-b<br>L-serine reversible transport via proton symport-b<br>thiamine-phosphate kinase-b

Table S3. Summary of overrepresented GO Terms. The columns are: GO term; p-value; Count: number of genes in the query data set annotated to a certain GO term; Size: the total number of genes related to the respective GO Term; Term: biological process. For cluster 1 under cold stress no GO Terms were found.

GOBPID	p-value	Count	Size	Term
Cold				
Cluster 2				
GO:0009063	1.9746e-06	4	75	cellular amino acid catabolic process
GO:0016054	2.8415e-06	4	82	organic acid catabolic process
GO:0044106	0.0004	4	272	cellular amine metabolic process
GO:0019752	0.0007	4	325	carboxylic acid metabolic process
GO:0042180	0.0007	4	325	cellular ketone metabolic process
GO:0009056	0.0027	4	448	catabolic process
GO:0006086	0.0061	1	3	acetyl-CoA biosynthetic process from pyruvate
GO:0009436	0.0122	1	6	glyoxylate catabolic process
GO:0006081	0.0322	1	16	cellular aldehyde metabolic process
GO:0006096	0.0362	1	18	glycolysis
GO:0006099	0.0402	1	20	tricarboxylic acid cycle
GO:0009109	0.0402	1	20	coenzyme catabolic process
GO:0006084	0.0441	1	22	acetyl-CoA metabolic process
Cluster 3				
GO:0016052	0.0004	7	276	carbohydrate catabolic process
GO:0009088	0.0009	2	8	threonine biosynthetic process
GO:0009052	0.0012	2	9	pentose-phosphate shunt, non-oxidative branch
GO:0006740	0.0015	2	10	NADPH regeneration
GO:0009152	0.0050	2	18	purine ribonucleotide biosynthetic process
GO:0009259	0.0050	2	18	ribonucleotide metabolic process
GO:0055086	0.0062	4	131	nucleobase, nucleoside and nucleotide metabolic process
GO:0006733	0.0111	2	27	oxidoreduction coenzyme metabolic process
GO:0046496	0.0111	2	27	nicotinamide nucleotide metabolic process
GO:0072524	0.0111	2	27	pyridine-containing compound metabolic process
GO:0006007	0.0119	2	28	glucose catabolic process
GO:0046365	0.0119	2	28	monosaccharide catabolic process
GO:0006163	0.0173	2	34	purine nucleotide metabolic process
GO:0072522	0.0203	2	37	purine-containing compound biosynthetic process
GO:0009117	0.0213	2	45	nucleotide metabolic process
GO:0044282	0.0242	3	107	small molecule catabolic process
GO:0009090	0.0243	1	4	homoserine biosynthetic process
GO:0009066	0.0293	2	45	aspartate family amino acid metabolic process
GO:0044238	0.0350	12	1485	primary metabolic process
GO:0019318	0.0357	2	50	hexose metabolic process
Cluster 4				
GO:0006094	0.0001	2	13	gluconeogenesis
GO:0006096	0.0002	2	18	glycolysis
GO:0019320	0.0006	2	28	hexose catabolic process
GO:0044275	0.0006	2	28	cellular carbohydrate catabolic process
GO:0046164	0.0006	2	28	alcohol catabolic process
GO:0046364	0.0006	2	28	monosaccharide biosynthetic process
GO:0009056	0.0118	3	448	catabolic process
GO:0016051	0.0168	2	151	carbohydrate biosynthetic process
Cluster 5				
GO:0015949	0.0003	4	61	nucleobase, nucleoside and nucleotide interconversion
GO:0016052	0.0004	7	276	carbohydrate catabolic process
GO:0044238	0.0350	12	1485	primary metabolic process
Cluster 6				
GO:0034654	0.0011	2	65	nucleobase, nucleoside,nucleotide and nucleic acid biosynthetic process
GO:0006753	0.0016	2	79	nucleoside phosphate metabolic process
GO:0009435	0.0173	1	17	NAD biosynthetic process
GO:0019363	0.0173	1	17	pyridine nucleotide biosynthetic process
GO:0009152	0.0183	1	18	purine ribonucleotide biosynthetic process
GO:0009259	0.0183	1	18	ribonucleotide metabolic process

Continued on next page

Table S3 – continued from previous page

GO:0044283	0.0216	2	289	small molecule biosynthetic process
GO:0009165	0.0231	1	45	nucleotide biosynthetic process
GO:0006733	0.0273	1	27	oxidoreduction coenzyme metabolic process
GO:0046496	0.0273	1	27	nicotinamide nucleotide metabolic process
GO:0072524	0.0273	1	27	pyridine-containing compound metabolic process
GO:0006163	0.0344	1	34	purine nucleotide metabolic process
GO:0072522	0.0374	1	37	purine-containing compound biosynthetic process
Cluster 7				
GO:0046394	1.4533e-05	12	185	carboxylic acid biosynthetic process
GO:0009090	1.5610e-05	3	4	homoserine biosynthetic process
GO:0044283	6.4233e-05	14	289	small molecule biosynthetic process
GO:0009309	7.0043e-05	11	181	amine biosynthetic process
GO:0009069	0.0003	4	21	serine family amino acid metabolic process
GO:0043648	0.0007	5	46	dicarboxylic acid metabolic process
GO:0006571	0.0025	2	5	tyrosine biosynthetic process
GO:0015949	0.0026	5	61	nucleobase,nucleoside and nucleotide interconversion
GO:0009073	0.0032	3	19	aromatic amino acid family biosynthetic process
GO:0043436	0.0034	12	325	oxoacid metabolic process
GO:0009094	0.0051	2	7	L-phenylalanine biosynthetic process
GO:0009088	0.0068	2	8	threonine biosynthetic process
GO:0009067	0.0069	3	27	aspartate family amino acid biosynthetic process
GO:0009086	0.0154	2	12	methionine biosynthetic process
GO:0015749	0.0163	1	1	monosaccharide transport
GO:0015753	0.0163	1	1	D-xylose transport
GO:0006725	0.0186	4	64	cellular aromatic compound metabolic process
GO:0000096	0.0335	2	18	sulfur amino acid metabolic process
GO:0006553	0.0335	2	18	lysine metabolic process
GO:0009089	0.0335	2	18	lysine biosynthetic process via diaminopimelate
GO:0043603	0.0335	2	18	cellular amide metabolic process
GO:0006520	0.0359	7	224	cellular amino acid metabolic process
GO:0006099	0.0408	2	20	tricarboxylic acid cycle
GO:0009109	0.0408	2	20	coenzyme catabolic process
GO:0044272	0.0437	3	49	sulfur compound biosynthetic process
GO:0006084	0.0486	2	22	acetyl-CoA metabolic process
Cluster 8				
GO:0006526	0.0004	2	24	arginine biosynthetic process
GO:0009064	0.0020	2	52	glutamine family amino acid metabolic process
GO:0044271	0.0028	3	279	cellular nitrogen compound biosynthetic process
GO:0008652	0.0195	2	163	cellular amino acid biosynthetic process
GO:0009228	0.0213	1	14	thiamine biosynthetic process
GO:0042723	0.0213	1	14	thiamine-containing compound metabolic process
GO:0044281	0.0218	3	549	small molecule metabolic process
GO:0016053	0.0249	2	185	organic acid biosynthetic process
Cluster 9				
GO:0015949	0.0029	3	61	nucleobase,nucleoside and nucleotide interconversion
GO:0044281	0.0068	7	549	small molecule metabolic process
GO:0006006	0.0224	2	47	glucose metabolic process
GO:0006564	0.0302	1	6	L-serine biosynthetic process
GO:0009088	0.0401	1	8	threonine biosynthetic process
GO:0016052	0.0402	4	276	carbohydrate catabolic process
GO:0005996	0.0410	2	65	monosaccharide metabolic process
Heat				
Cluster 1				
GO:0006094	0.0004	2	13	gluconeogenesis
GO:0006096	0.0007	2	18	glycolysis
GO:0019320	0.0019	2	28	hexose catabolic process
GO:0044275	0.0019	2	28	cellular carbohydrate catabolic process
GO:0046164	0.0019	2	28	alcohol catabolic process
GO:0046364	0.0019	2	28	monosaccharide biosynthetic process
GO:0034404	0.0101	2	65	nucleobase, nucleoside and nucleotide biosynthetic process
GO:0006753	0.0147	2	79	nucleoside phosphate metabolic process
GO:0044283	0.0384	2	224	small molecule biosynthetic process
GO:0009435	0.0426	1	17	NAD biosynthetic process
GO:0019363	0.0426	1	17	pyridine nucleotide biosynthetic process

Continued on next page

Table S3 – continued from previous page

GO:0009152	0.0450	1	18	purine ribonucleotide biosynthetic process
GO:0009259	0.0450	1	18	ribonucleotide metabolic process
GO:0018130	0.0479	2	147	heterocycle biosynthetic process
Cluster 2				
GO:0009063	1.9746e-06	4	75	cellular amino acid catabolic process
GO:0016054	2.8415e-06	4	82	organic acid catabolic process
GO:0044106	0.0004	4	272	cellular amine metabolic process
GO:0019752	0.0007	4	325	carboxylic acid metabolic process
GO:0042180	0.0007	4	325	cellular ketone metabolic process
GO:0009056	0.0027	4	448	catabolic process
GO:0006086	0.0061	1	3	acetyl-CoA biosynthetic process from pyruvate
GO:0009436	0.0122	1	6	glyoxylate catabolic process
GO:0006081	0.0322	1	16	cellular aldehyde metabolic process
GO:0006096	0.0362	1	18	glycolysis
GO:0006099	0.0402	1	20	tricarboxylic acid cycle
GO:0009109	0.0402	1	20	coenzyme catabolic process
GO:0006084	0.0441	1	22	acetyl-CoA metabolic process
Cluster 3				
GO:0016052	0.0008	7	276	carbohydrate catabolic process
GO:0009052	0.0014	2	9	pentose-phosphate shunt, non-oxidative branch
GO:0006740	0.0018	2	10	NADPH regeneration
GO:0009152	0.0058	2	18	purine ribonucleotide biosynthetic process
GO:0009259	0.0058	2	18	ribonucleotide metabolic process
GO:0055086	0.0085	4	131	nucleobase, nucleoside and nucleotide metabolic process
GO:0006526	0.0103	2	24	arginine biosynthetic process
GO:0006733	0.0130	2	27	oxidoreduction coenzyme metabolic process
GO:0046496	0.0130	2	27	nicotinamide nucleotide metabolic process
GO:0072524	0.0130	2	27	pyridine-containing compound metabolic process
GO:0006007	0.0139	2	28	glucose catabolic process
GO:0046365	0.0139	2	28	monosaccharide catabolic process
GO:0006163	0.0202	2	34	purine nucleotide metabolic process
GO:0072522	0.0237	2	37	purine-containing compound biosynthetic process
GO:0009117	0.0256	2	45	nucleotide metabolic process
GO:0044238	0.0264	13	1485	primary metabolic process
GO:0044282	0.0302	3	107	small molecule catabolic process
GO:0044283	0.0313	5	289	small molecule biosynthetic process
GO:0019318	0.0415	2	50	hexose metabolic process
GO:0009064	0.0446	2	52	glutamine family amino acid metabolic process
Cluster 4				
GO:0006596	6.2969e-09	4	19	polyamine biosynthetic process
GO:0006576	5.8420e-08	4	32	cellular biogenic amine metabolic process
GO:0009310	9.3790e-06	4	110	amine catabolic process
GO:0009309	7.0267e-05	4	181	amine biosynthetic process
GO:0034641	0.0433	4	896	cellular nitrogen compound metabolic process
GO:0044249	0.0469	4	914	cellular biosynthetic process
Cluster 5				
GO:0009090	0.0020	1	4	homoserine biosynthetic process
GO:0009088	0.0041	1	8	threonine biosynthetic process
GO:0009086	0.0061	1	12	methionine biosynthetic process
GO:0000096	0.0092	1	18	sulfur amino acid metabolic process
GO:0006553	0.0092	1	18	lysine metabolic process
GO:0009089	0.0092	1	18	lysine biosynthetic process via diaminopimelate
GO:0009069	0.0107	1	21	serine family amino acid metabolic process
GO:0043648	0.0234	1	46	dicarboxylic acid metabolic process
GO:0044272	0.0250	1	49	sulfur compound biosynthetic process
Cluster 6				
GO:0009152	0.0092	1	18	purine ribonucleotide biosynthetic process
GO:0009259	0.0092	1	18	ribonucleotide metabolic process
GO:0006163	0.0173	1	34	purine nucleotide metabolic process
GO:0072522	0.0189	1	37	purine-containing compound biosynthetic process
GO:0009165	0.0316	1	62	nucleotide biosynthetic process
GO:0034654	0.0331	1	65	nucleobase, nucleoside, nucleotide and nucleic acid biosynthetic process
GO:0006753	0.0403	1	79	nucleoside phosphate metabolic process

Continued on next page

Table S3 – continued from previous page

Cluster 7				
GO:0046394	1.4533e-05	12	185	carboxylic acid biosynthetic process
GO:0009090	1.5610e-05	3	4	homoserine biosynthetic process
GO:0044283	6.4233e-05	14	289	small molecule biosynthetic process
GO:0009309	7.0043e-05	11	181	amine biosynthetic process
GO:0009069	0.0003	4	21	serine family amino acid metabolic process
GO:0043648	0.0007	5	46	dicarboxylic acid metabolic process
GO:0006571	0.0025	2	5	tyrosine biosynthetic process
GO:0015949	0.0026	5	61	nucleobase, nucleoside and nucleotide interconversion
GO:0009073	0.0032	3	19	aromatic amino acid family biosynthetic process
GO:0043436	0.0034	12	325	oxoacid metabolic process
GO:0009094	0.0051	2	7	L-phenylalanine biosynthetic process
GO:0009088	0.0068	2	8	threonine biosynthetic process
GO:0009067	0.0069	3	27	aspartate family amino acid biosynthetic process
GO:0009086	0.0154	2	12	methionine biosynthetic process
GO:0015749	0.0163	1	1	monosaccharide transport
GO:0015753	0.0163	1	1	D-xylose transport
GO:0006725	0.0186	4	64	cellular aromatic compound metabolic process
GO:0000096	0.0335	2	18	sulfur amino acid metabolic process
GO:0006553	0.0335	2	18	lysine metabolic process
GO:0009089	0.0335	2	18	lysine biosynthetic process via diaminopimelate
GO:0043603	0.0335	2	18	cellular amide metabolic process
GO:0006520	0.0359	7	224	cellular amino acid metabolic process
GO:0006099	0.0408	2	20	tricarboxylic acid cycle
GO:0009109	0.0408	2	20	coenzyme catabolic process
GO:0044272	0.0437	3	49	sulfur compound biosynthetic process
GO:0006084	0.0486	2	22	acetyl-CoA metabolic process
Cluster 8				
GO:0016052	4.56285e-05	6	276	carbohydrate catabolic process
Cluster 9				
GO:0015949	0.0001	6	61	nucleobase, nucleoside and nucleotide interconversion
GO:0006571	0.0018	2	5	tyrosine biosynthetic process
GO:0009073	0.0019	3	19	aromatic amino acid family biosynthetic process
GO:0043648	0.0031	4	46	dicarboxylic acid metabolic process
GO:0044283	0.0034	10	289	small molecule biosynthetic process
GO:0009094	0.0037	2	7	L-phenylalanine biosynthetic process
GO:0044281	0.0097	7	260	small molecule metabolic process
GO:0006725	0.0103	4	64	cellular aromatic compound metabolic process
GO:0009061	0.0104	6	141	anaerobic respiration
GO:0015749	0.0138	1	1	monosaccharide transport
GO:0015753	0.0138	1	1	D-xylose transport
GO:0006099	0.0298	2	20	tricarboxylic acid cycle
GO:0009109	0.0298	2	20	coenzyme catabolic process
GO:0009309	0.0325	6	181	amine biosynthetic process
GO:0006084	0.0356	2	22	acetyl-CoA metabolic process
GO:0046394	0.0357	6	185	carboxylic acid biosynthetic process
GO:0015980	0.0374	6	187	energy derivation by oxidation of organic compounds

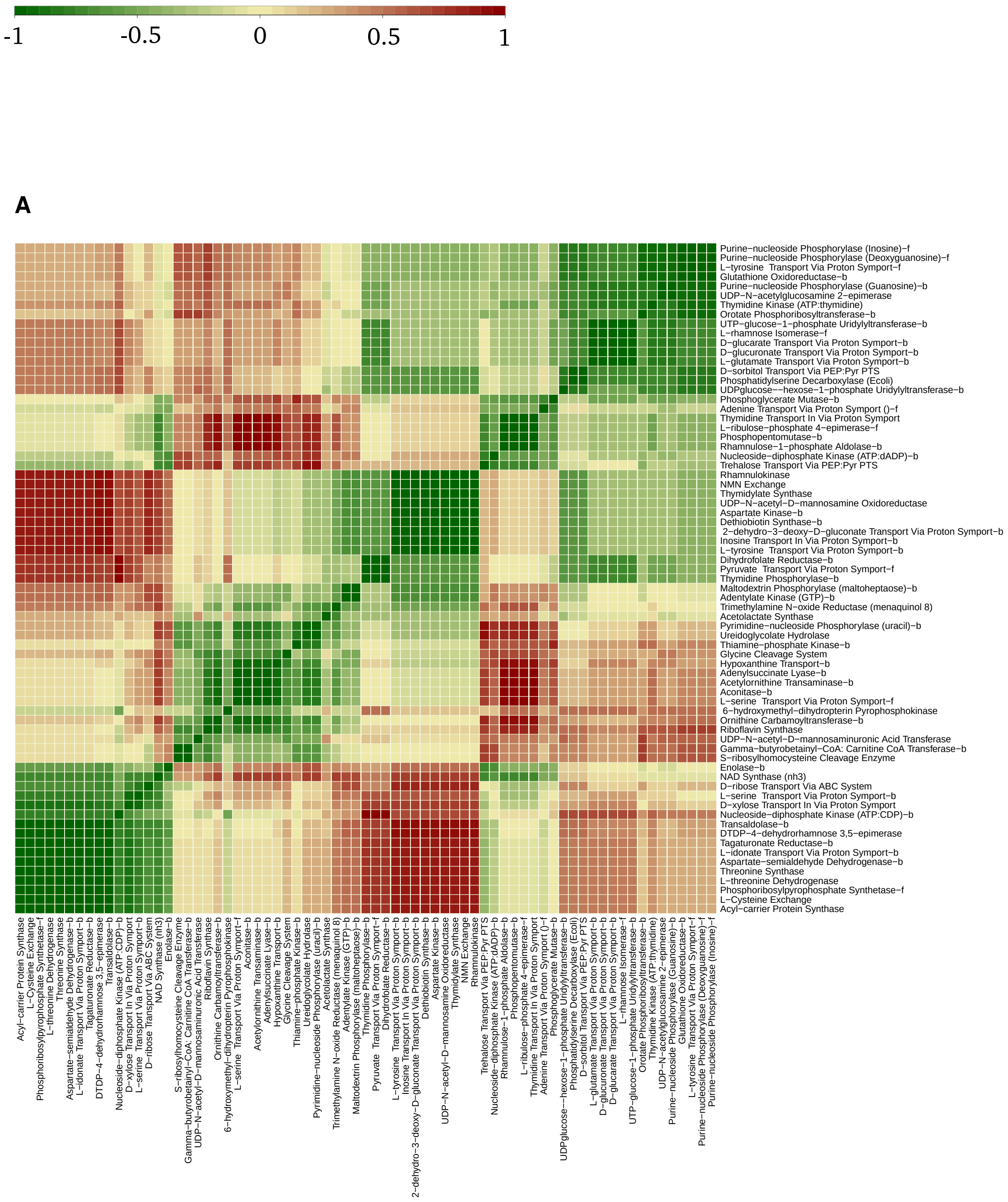

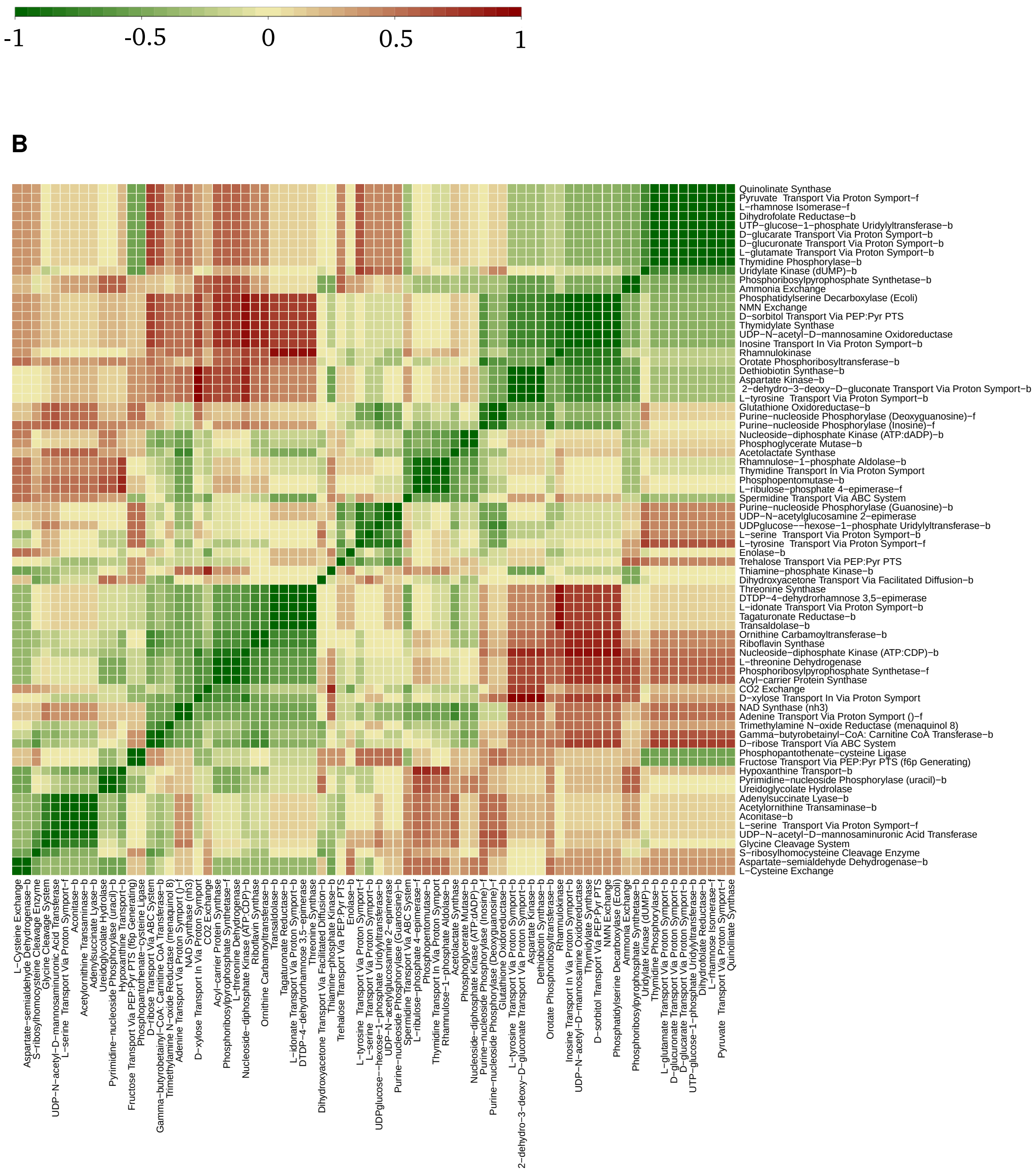

Figure S1. Heatmaps of the Kendall correlation for fractional appearance profiles for selected reactions. (A) cold shock, (B) heat shock. Green fields indicate a positive correlation, yellow no correlation and red a negative correlation between profiles of fractional appearance in EFMs over time. The selected reactions are the set union of the 50 most in EFMs occurring reactions over all time-points.

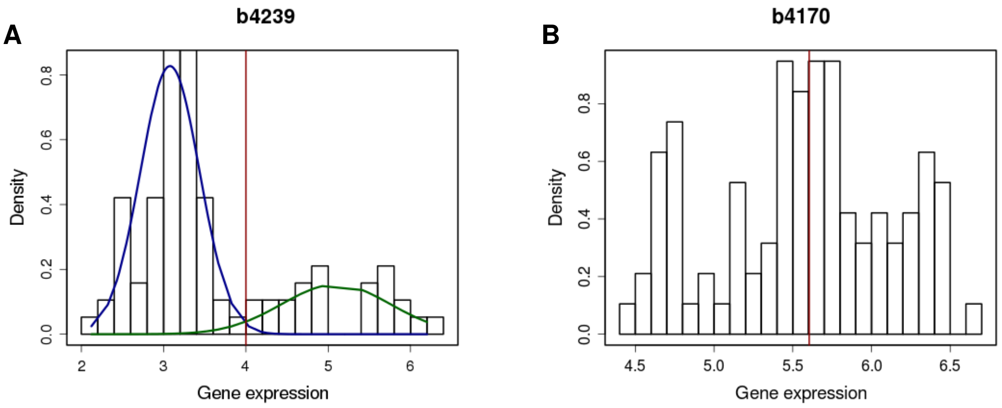

Figure S2. Histogram of gene expression values for two selected genes. Distributions over three replicates and five different environmental conditions for 7 (5) time-points. (A) gene, which expression values show bimodality and (B) without bimodal distribution. The vertical line indicates the gene-specific threshold, which was determined by calculating the intersection of both fitted curves or by calculating the median, respectively.

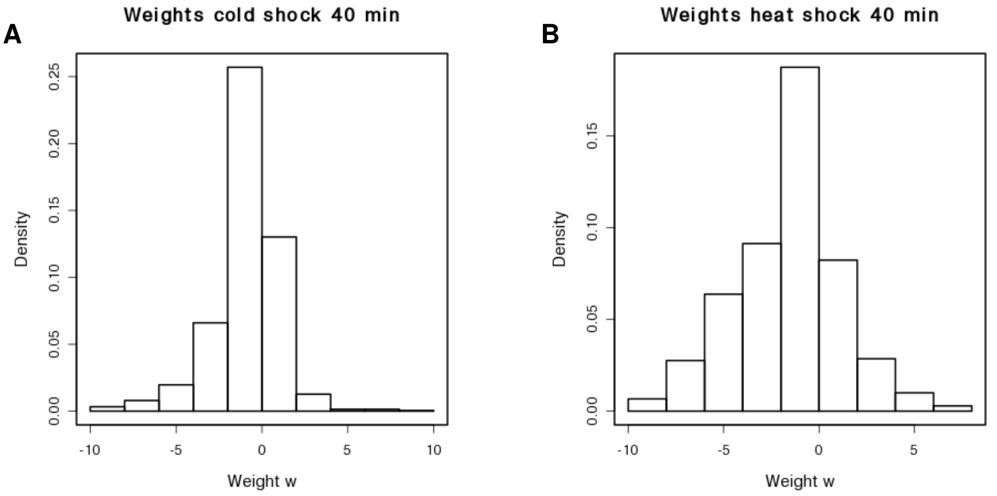

s

Figure S3. Distributions of time- and condition-specific weights. Distributions of weights for the metabolic reactions for (A) cold and (B) heat shock after 40 min. For reasons of clearness, outliers are removed and only weights in the interval between -10 and 10 are depicted.

References

1. Jozefczuk, S, Klie, S, Catchpole, G, Szymanski, J, Cuadros-Inostroza, A, Steinhauser, D, Selbig, J, & Willmitzer, L. (2010) *Molecular systems biology* **6**, 364.

2. R Development Core Team. (2011) *R: A Language and Environment for Statistical Computing* (R Foundation for Statistical Computing, Vienna, Austria). ISBN 3-900051-07-0.

3. Smyth, G. K. (2004) *Statistical applications in genetics and molecular biology* **3**, Article3.

4. Benaglia, T, Chauveau, D, Hunter, D. R, & Young, D. (2009) *Journal of Statistical Software* **32**, 1–29.

5. Reed, J. L, Vo, T. D, Schilling, C. H, & Palsson, B. O. (2003) *Genome biology* **4**, R54.

6. Orth, J. D, Thiele, I, & Palsson, B. O. (2010) *Nature biotechnology* **28**, 245–8.

7. Feist, A. M & Palsson, B. O. (2010) *Current opinion in microbiology* **13**, 349–344.

8. Schuetz, R, Kuepfer, L, & Sauer, U. (2007) *Molecular systems biology* **3**, 119.

9. Schuster, S & Hilgetag, C. (1994) *Journal of Biological Systems* **2**, 165–182.

10. Vanderbei, R. J. (1996) *Linear Programming: Foundations and Extensions.* (Kluwer Academic, Boston, USA), p. 416.

11. Arroyo, J & Galiana, F. (2005) *IEEE Transactions on Power Systems* **20**, 789–797.

12. Burgard, A. P, Pharkya, P, & Maranas, C. D. (2003) *Biotechnology and bioengineering* **84**, 647–57.

13. MATLAB. (2011) version 7.12(r2011a).

14. Keating, S. M, Bornstein, B. J, Finney, A, & Hucka, M. (2006) *Bioinformatics (Oxford, England)* **22**, 1275–7.

15. Holmström, K. (1998) Tomlab - a general purpose, open matlab environment for research and teaching in optimization.

16. Terzer, M & Stelling, J. (2008) *Bioinformatics (Oxford, England)* **24**, 2229–35.

17. Maechler, M, Rousseeuw, P, Struyf, A, & Hubert, M. (2005) Cluster analysis basics and extensions.

18. Rousseeuw, P. J. (1987) **20**, 53–65.

19. Falcon, S & Gentleman, R. (2007) *Bioinformatics* **23**, 257–8.

20. Carlson, M, Falcon, S, Pages, H, & Li, N. (2011) *org.EcK12.eg.db: Genome wide annotation for E coli strain K12.* R package version 2.5.0.
